# Chaos Quantum-Behaved Cat Swarm Optimization Algorithm and Its Application in the PV MPPT

**DOI:** 10.1155/2017/1583847

**Published:** 2017-10-17

**Authors:** Xiaohua Nie, Wei Wang, Haoyao Nie

**Affiliations:** ^1^Information Engineering School, Nanchang University, Nanchang, Jiangxi Province 330031, China; ^2^Economics Management School, Nanchang University, Nanchang, Jiangxi Province 330031, China

## Abstract

Cat Swarm Optimization (CSO) algorithm was put forward in 2006. Despite a faster convergence speed compared with Particle Swarm Optimization (PSO) algorithm, the application of CSO is greatly limited by the drawback of “premature convergence,” that is, the possibility of trapping in local optimum when dealing with nonlinear optimization problem with a large number of local extreme values. In order to surmount the shortcomings of CSO, Chaos Quantum-behaved Cat Swarm Optimization (CQCSO) algorithm is proposed in this paper. Firstly, Quantum-behaved Cat Swarm Optimization (QCSO) algorithm improves the accuracy of the CSO algorithm, because it is easy to fall into the local optimum in the later stage. Chaos Quantum-behaved Cat Swarm Optimization (CQCSO) algorithm is proposed by introducing tent map for jumping out of local optimum in this paper. Secondly, CQCSO has been applied in the simulation of five different test functions, showing higher accuracy and less time consumption than CSO and QCSO. Finally, photovoltaic MPPT model and experimental platform are established and global maximum power point tracking control strategy is achieved by CQCSO algorithm, the effectiveness and efficiency of which have been verified by both simulation and experiment.

## 1. Introduction

Solar energy is widely used due to the advantages of renewable and nonpolluting. Global maximum power point tracking (GMPPT) is one of the basic means to improve the overall efficiency of photovoltaic power generation system. However, the traditional maximum power point tracking (MPPT) algorithm is often ineffective because of the output power curve with multipeak problem in complex shade conditions. The problem results in efficiency reducing of photovoltaic power generation [1, 2]. Many scholars have carried out massive researches to solve these questions. Many optimization algorithms are used to realize global maximum power point tracking and achieve good results, like Particle Swarm Optimization (PSO) algorithm, evolutionary algorithm, fuzzy logic control algorithm, neural network control algorithm, chaos search algorithm, and so on [2–9].

The Cat Swarm Optimization (CSO) algorithm was proposed by Chu et al. in 2006 [10]. The experimental results showed that the CSO algorithm could find the global optimal solution in a short time, and the CSO algorithm is better than the PSO algorithm in the convergence speed and nonlinear optimization of the local extremum [11–18]. However, the CSO algorithm has the shortcoming of “premature convergence” in the practical application. If the number of iteration is increased continuously, it will cause the convergence time to multiply while the accuracy of the optimal value is not significantly improved. The same problems also occur in the PSO algorithm. A novel chaotic quantum-behaved PSO algorithm is proposed for solving nonlinear system of equations in [19]. Different chaotic maps are introduced to enhance the effectiveness and robustness of the algorithm. The comparison of results reveals that the proposed algorithm can cope with the highly nonlinear problems and outperform other algorithms. The Hybrid Chaotic Quantum-behaved Particle Swarm Optimization (HCQPSO) algorithm is used for thermal design of plate fin heat exchangers in [20]. The HCPQSO algorithm successfully combines a variant of Quantum-behaved Particle Swarm Optimization with efficient local search mechanisms to yield better results in terms of solution accuracy and convergence rate. It is also observed that the proposed algorithm successfully converges to optimum configuration with a higher accuracy. A chaotic improved Particle Swarm Optimization algorithm is proposed for photovoltaic MPPT in [21]. An improved cuckoo search (ICS) algorithm is proposed to establish the parameters of chaotic systems in [22]. The numerical results demonstrate that the algorithm can estimate parameters with high accuracy and reliability. An evolutionary approach of parking space guidance is proposed based upon a novel Chaotic Particle Swarm Optimization (CPSO) algorithm in [23]. The Chaotic Firefly algorithm using tent maps is proposed for optimally coordinating the relays in [24]. Chaos theory has been incorporated to prevent the search process from being trapped in local minima by modifying the concept of random movement factor variable.

In this paper, a Chaos Quantum-behaved Cat Swarm Optimization (CQCSO) algorithm is proposed. The cat with the quantum behavior has no definite trajectory in tracking and has the possibility of getting rid of the local optimal point with large disturbance. Since there is only position vector in the control parameter, with no velocity vector, and the convergence time is shortened, then, the tent chaotic map is introduced to change the cat position information in the iteration process, the “premature convergence” problem of the CSO algorithm is avoided, and the precision of searching is further improved. In Section 2, the CQCSO algorithm is tested in five nonlinear function curves with multiple local extreme points. The results show that the proposed CQCSO algorithm has high tracking precision and fast convergence speed. Moreover, the proposed CQCSO algorithm has the ability to adapt to complex and different curves. In Section 3, the proposed CQCSO algorithm is applied to PV MPPT control, and a multipeak MPPT control strategy based on CQCSO algorithm is proposed. The simulation and experimental results show that the proposed control strategy more efficiently tracks the global maximum power points.

## 2. Chaos Quantum-Behaved Cat Swarm Optimization (CQCSO) Algorithm

### 2.1. QCSO Algorithm

The CSO algorithm is a kind of swarm intelligence algorithms based on the cat's living habits and foraging. It is superior to the PSO in searching accuracy and convergence time [12–19]. It is widely used to solve the optimization problem. In the CSO algorithm, the cat's position is regarded as a feasible solution to the optimization problem, and then the cat swarms are divided into two groups according to the mixture ratio (MR), which are the searching cat group and the tracking cat group. The searching cat group refers to the genetic algorithm to complete the cat's location update. The cats with the highest fitness value are selected to replace the current cat position by copying and mutating individuals. The tracking cat groups are similar to the PSO algorithm and use the cat's own speed and the current position information to update the position of the cat continuously, so that each individual can move closer to the global optimal solution. Although the search of the global optimal solution can be realized, there exists shortcoming of “premature convergence” problem like other swarm intelligent algorithms.

Quantum-behaved Cat Swarm Optimization (QCSO) algorithm is a combination of CSO algorithms and quantum mechanics. In the evolutionary process, each tracing cat has a *B*_*i*_-centered DELTA potential well, which makes each tracing cat converge to an attractor *B*_*i*_. It continuously updates the location of the cat by tracking individual extremes and global extremes, so that the cat's speed and location are uncertain. So, it can be distributed in a certain probability to search space at any position. It is possible to get rid of the local optimal points in disturbing environment. As a result, the QCSO algorithm can jump out of the local optimum and improve the accuracy of CSO algorithm.

The updated expression of individual position in quantum space is

(1) $$\begin{array}{c} X_{i}^{k+1}=B_{i}^{k}+ba_{k}\left|\;C_{}^{k}-X_{i}^{k}\;\right|\ln\left(\;r_{1}^{-1}\;\right), \end{array}$$

where

(2) Bik=r2Dik+1−r2Gk,b=−1,r3≤0.51,r3>0.5,ak=a1−a1−a2k′k′¯,C1k,C2k,…,Cdk=1m∑i=1mDi1k,∑i=1mDi2k,…,∑i=1mDidk,

where *m* is population size, *i* = 1,2,…, *m*, *d* is dimension, *k* is maximum number of iterations, $k=1,2,\ldots ,\bar{k}$, *k*′ is maximum number of traces, $k^{\prime }=1,2,\ldots ,\bar{k^{\prime }}$, *C*^*k*^ is the cat group optimal position center of the *k*th iteration, *X*_*i*_^*k*^ is the optimal position of the *i*th cat for the *k*th iteration, *D*_*i*_^*k*^ is the optimal position of the *i*th cat for the *k*th iteration, *G*^*k*^ is the global optimal position of the population at the *k*th iteration, *a*_*k*_ is the *k*th iteration expansion contraction factor, *a*_1_, *a*_2_ are the initial compression factor and the termination value, respectively, *a*_1_ = 1.0, *a*_2_ = 0.5, and *r*_1_, *r*_2_, *r*_3_ are random numbers with uniform distribution in the [0,1] interval.

### 2.2. CQCSO Algorithm

The QCSO algorithm improves the accuracy of the CSO algorithm, because the cat in the evolutionary process continues to move closer to the optimal position of the population, the diversity of the population gradually decreases, and it is easy to fall into the local optimum in the later stage. Chaos Quantum-behaved Cat Swarm Optimization (CQCSO) algorithm is proposed by introducing tent map for jumping out of local optimum in this paper.

The individual location update expression of tent map is as follows:

(3) $$\begin{array}{c} x_{i}^{k+1}=\left\{\begin{array}{cc} 2x_{i}^{k}, & 0\leq x_{i}^{k}\leq 0.5\\ 2\left(1-x_{i}^{k}\right), & 0.5<x_{i}^{k}\leq 1, \end{array}\right. \end{array}$$

where $x_{i}^{k}\in \left[\begin{array}{cc} 0 & 1 \end{array}\right]$, *i* = 1,2,…, *m*, $k=1,2,\ldots ,\bar{k}$. $x_{i}^{k}\in \left[\begin{array}{cc} 0 & 1 \end{array}\right]$ can be mutually mapped transformation with the chaotic variables $Y_{i}^{k}\in \left[\begin{array}{cc} a & b \end{array}\right]$ through

(4) xik=Yik−ab−a,

(5) Yik=a+xikb−a.

Detailed CQCSO algorithm steps are as follows.

**Step 1 .**

Initialize the cat swarm, set the population size *m*, the maximum iteration number $\overset{-}{k}$, and the mixture ratio (MR) of the cat optimization algorithm, and randomly initialize position of the cat population between [*a*  *b*]; it is expressed with the row vector *Y*.

**Step 2 .**

The fitness value of all cats in the population was calculated, and the cat with the greatest fitness was selected and recorded.

**Step 3 .**

According to MR, cats swarms are randomly grouped. MR represents the proportion of the number of cats in the tracking group in the entire cat population. MR is generally a smaller value to ensure that most of the cats in the swarm are in search mode and a few cats are in tracking mode.

**Step 4 .**

When the cat is in the search group, it replicates its position according to the size of seeking memory pool (SMP), executes the selection operator, updates SMP, and replaces the position of the current cat with the candidate point with the highest fitness value. In the end, the optimal value updating is completed. The cat of tracking group updates its own position information according to formula (1).

**Step 5 .**

The cat with the best fitness is recorded in the reserved populations.

**Step 6 .**

It is judged whether the termination condition is satisfied, and if so, the program ends and the optimal solution is output. If it is not satisfied, it is judged whether the position of the global optimal cat is the same after the *k*th and *k* − 1 iterations, and if not, Steps 3–6 are repeated. If it is the same, it means that it has fallen into local optimum and needs to deal with chaos. The mapping of the normal variable *Y*_*i*_^*k*^ is performed by using formula (4). The chaotic variables *x*_*i*_^*k*^ after mapping are in the range [0  1]. Chaos variable *x*_*i*_^*k*^ is mapped to get *x*_*i*_^*k*+1^ using formula (3). And then through formula (5), chaos variable *x*_*i*_^*k*+1^ mapping transformation, the next iteration of the conventional variables *Y*_*i*_^*k*+1^ is obtained. Steps 2–6 are repeated to optimize iteration.

### 2.3. Verification for CQCSO Algorithm

In order to verify the superiority of the CQCSO algorithm, five kinds of nonlinear functions with multiple local extremum peaks such as Schaffer, Shubert, Griewank, Rastrigrin, and Rosenbrock are compared for seeking optimization. In simulation, the total number of cat groups is set to 20; each function program runs 50 times.

**Figure 1:**
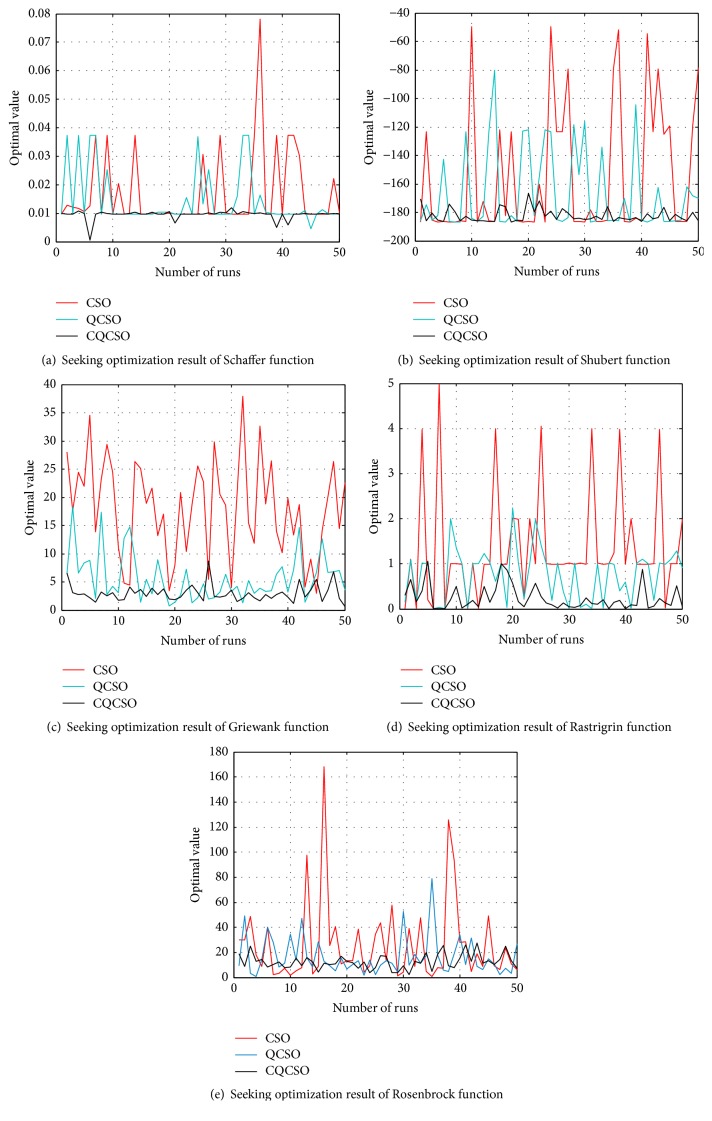
Seeking optimization results of five function.

*(1) Schaffer Function*

(6) $$\begin{array}{c} \min f\left(\;x_{1},x_{2}\;\right)=0.5+\frac{{\left(\sin\sqrt{x_{1}^{2}+x_{2}^{2}}\right)}^{2}-0.5}{{\left(1+0.001\left(x_{1}^{2}+x_{2}^{2}\right)\right)}^{2}}, \end{array}$$

where *x*_1_, *x*_2_ ∈ [−10,10]; Schaffer function is a two-dimensional complex function with numerous small points. The minimum value 0 is obtained at (0, 0). Because this function has strong concussion, it is hard to find the global optimal value. The seeking optimization result is obtained and shown in Figure 1(a).

*(2) Shubert Function*

(7) min⁡fx,y=∑i=15icos⁡i+1x+i×∑i=15icos⁡i+1y+i,

where *x*, *y* ∈ [−10,10]; Shubert function is a two-dimensional complex function with 760 local extrema points. The global minimum value −186.7309 is obtained at (−1.42513, 0.80032). The seeking optimization result is obtained and shown in Figure 1(b).

*(3) Griewank Function*

(8) $$\begin{array}{c} \min f\left(\;x_{i}\;\right)=\overset{D}{\underset{i=1}{\sum }}\frac{x_{i}^{2}}{4000}-\overset{D}{\underset{i=1}{\prod }}\cos\left(\;\frac{x_{i}}{\sqrt{i}}\;\right)+1, \end{array}$$

where *x*_*i*_ ∈ [−600,600]; Griewank function has many local minimums whose numbers are related to the dimension. The global minimum value 0 is obtained at (*x*_1_, *x*_2_,…, *x*_*n*_) = (0,0,…, 0). Griewank function is a typical nonlinear multimodal function. It is usually considered to be a complex multimodal problem that is difficult to handle by the optimization algorithm. The function dimension *D* is set to 3. The seeking optimization result is obtained and shown in Figure 1(c).

*(4) Rastrigrin Function*

(9) $$\begin{array}{c} \min f\left(\;x_{i}\;\right)=\overset{D}{\underset{i=1}{\sum }}\left[\;x_{i}^{2}-10\cos\left(\;2\pi x_{i}\;\right)+10\;\right], \end{array}$$

where *x*_*i*_ ∈ [−5.12,5.12]; Rastrigrin function is a multimodal function with about 10D local minima. The global minimum value 0 is obtained at (*x*_1_, *x*_2_,…, *x*_*n*_) = (0,0,…, 0). Since its peak shape appears to fluctuate undulatingly, it is difficult to find the global optimal value. The function dimension *D* is set to 3. The seeking optimization result is obtained and shown in Figure 1(d).

*(5) Rosenbrock Function*

(10) $$\begin{array}{c} \min f\left(\;x_{i}\;\right)=\overset{D-1}{\underset{i=1}{\sum }}\left[\;100{\left(\;x_{i}^{2}-x_{i+1}\;\right)}^{2}+{\left(\;x_{i}-1\;\right)}^{2}\;\right], \end{array}$$

where *x*_*i*_ ∈ [−2.048,2.048]; the global optimal point of Rosenbrock function is located in a smooth, narrow parabolic valley. It is difficult to distinguish the search direction. The minimum value 0 is obtained at (*x*_1_, *x*_2_,…, *x*_*n*_) = (1,1,…, 1). The function dimension *D* is set to 4. The seeking optimization result is obtained and shown in Figure 1(e).

The seeking optimization of five functions is simulated 50 times. The simulation results are summed up in Table 1, where TIME is the average convergence time, BEST is the average optimum value, and STD is standard deviation of the optimal values in Figures 1(a)–1(e).

The Shubert function is used as an example. From Figure 1(b) and Table 1, the optimal value error obtained by CSO, QCSO, and CQCSO algorithm is 17.31%, 10.99%, and 2.43%, respectively, compared with the true value −186.7309. the precision of CQCSO algorithm is highest. In the convergence time, the QCSO algorithm shortens 0.0301 seconds compared with the CSO algorithm; then the CQCSO algorithm shortens 0.002 seconds compared with the QCSO algorithm. Therefore, simulation results show that the CQCSO algorithm can not only effectively solve the “premature convergence” problem of the CSO algorithm, but also improve the optimal solution accuracy and shorten the convergence time.

## 3. CQCSO Algorithm Application in PV MPPT

### 3.1. MPPT Flowchart Based on CQCSO Algorithm

The* P-V* characteristic curve function for photovoltaic cell can be gotten as follows [2–9]:

(11) $$\begin{array}{c} P_{L}=V_{L}\times I_{sc}\times \left[\;1-C_{1}\exp\left(\;\frac{V_{L}}{C_{2}\times V_{oc}}\;\right)\;\right], \end{array}$$

where *I*_sc_, *V*_oc_, *C*_1_, and *C*_2_ are the manufacturer-given parameters of photovoltaic cells. The output powers *P*_*L*_ change while output voltages *V*_*L*_ are adjusted. In this paper, the output voltages *V*_*L*_ are proportional to the duty cycles which are output by controller.

The* P-V* characteristic curve's fitness function (12) for photovoltaic array can be gotten from (11).

(12) $$\begin{array}{c} P_{array}=N_{s}\times N_{p}\times P_{L}, \end{array}$$

where *N*_*s*_ are the numbers of photovoltaic cell in series and *N*_*p*_ are the numbers of parallel photovoltaic cell strings.

**Figure 2:**
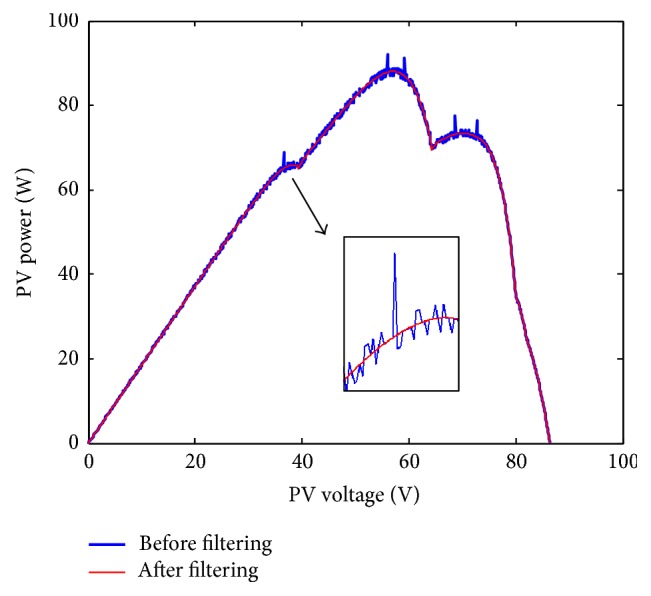
*P-V* characteristics of PV array under complex application environments.

While the cells in array are partially shaded, the photovoltaic array's* P-V* characteristic curve can be gotten as shown in Figure 2. The* P-V* curve shows the multipeak characteristic with measurement interference. It is one nonlinear function seeking optimization with multiple local extreme points. In order to track the maximum power point better, the recursive least squares method is used to previously filter in real time the* P-V* characteristic curve under the local shading condition before searching optimization.

**Figure 3:**
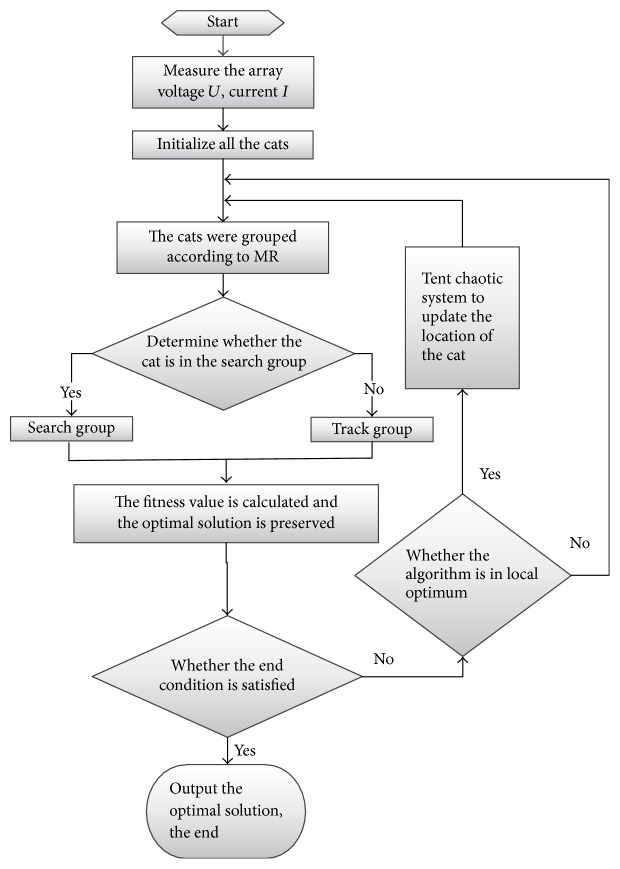
PV MPPT flowchart based on CQCSO algorithm.

PV MPPT control strategy flowchart based on CQCSO algorithm is shown in Figure 3. The position of each cat *X*_*i*_ is defined as the array output voltage value *U*_*i*_, and the fitness value is the array output power value.

### 3.2. Simulation Results

**Figure 4:**
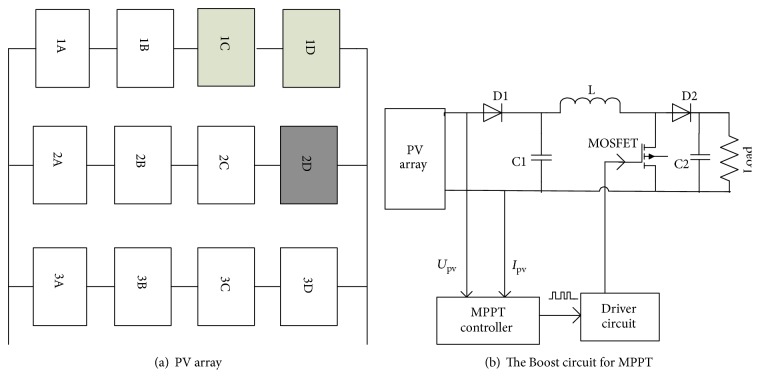
Configuration of PV array and circuit for MPPT.

In this paper, the MPPT control system is consisting of PV array, Boost circuit, and MPPT controller. PV array is composed of 3 × 4 PV cells. The array structure is shown in Figure 4(a). The detailed parameters of PV modules are given in Table 2. In Figure 4(a), the light irradiation of 1C and 1D PV cell is 700 W/m^2^, the light irradiation of the 2D PV cell is 100 W/m^2^, the light irradiation of the rest of the PV cells is 1000 W/m^2^, and the reference temperature of all PV cells is 25°C. The Boost circuits for MPPT are shown as Figure 4(b).

**Figure 5:**
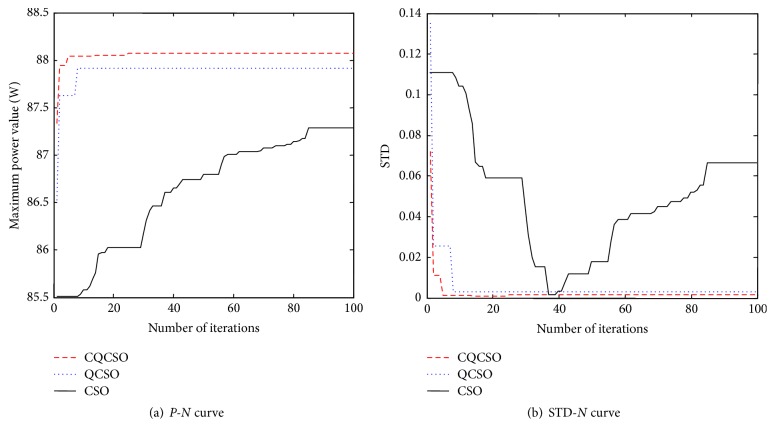
PV MPPT simulation curve.

The total number of cat groups is set to 20. The maximum number of iterations is 100. The PV global maximum power point tracking* P-N* (maximum power value power-number of iterations) curve and the STD-*N* (standard deviation-number of iterations) curve are obtained as shown in Figure 5. The simulation results show that the CQCSO algorithm can be applied to the maximum power point tracking of PV multipeak curve, and the global maximum power point is the best among the three algorithms (CSO, QCSO, and CQCSO).

Tent chaos is introduced into PSO algorithm to obtain CPSO algorithm [21]. CPSO algorithm is applied to maximum power point tracking in solar photovoltaic system. We compare the performances of the CSO, QCSO, CQCSO, PSO, and CPSO algorithms as shown in Table 3. The total number of cat groups is set to 10. The packet rate MR is 0.2. The maximum number of iterations is 100. The mean convergence time (Time), power minimum (Min), power maximum (Max), power mean (Mean), and standard deviation (STD) of 100 runs are summarized.

From Table 3, it is not difficult to find that CSO algorithm has an average convergence time of 0.00291 s, the average value obtained is 87.72 W, while the average run time of the PSO algorithm is 0.00431 s, and the average optimal value is 87.66 W. CSO and PSO algorithm are of premature convergence, and the convergence time is longer. These would lead to a significant reduction in the efficiency of photovoltaic power generation.

CPSO algorithm can effectively solve the PSO algorithm premature convergence problem, and the convergence time becomes shorter. The convergence time of QCSO algorithm is shortened from 0.00291 s to 0.00282 s compared with the convergence time of CSO algorithm, and the average power value increased from 87.72 W to 87.89 W. The CQCSO algorithm presented in this paper is the maximum power average of 88.02 using the shortest convergence time of 0.00268 s and the minimum STD value. Therefore, the simulation results show that the proposed CQCSO algorithm has high efficiency in the photovoltaic MPPT.

### 3.3. Experiment Results

**Figure 6:**
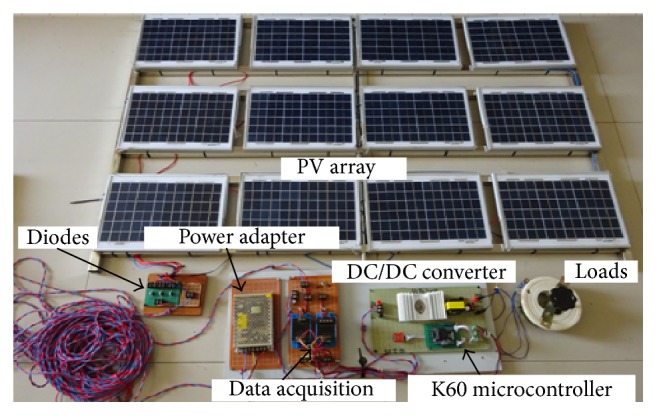
Experimental test platform.

In order to verify the validity of the proposed CQCSO algorithm in the MPPT control and that the CQCSO algorithm is more efficient than QCSO algorithm and CSO algorithm, the small-scale MPPT control system based on the K60 microcontroller is built and shown in Figure 6, which is established in accordance with the configuration shown in Figure 4.

The MPPT controller adopts MK60-DN512VLL10 chip, the voltage and current are real-timely sampled by Hall sensor and then are sent to the K60 microcontroller, and the data will be calculated to get the right duty cycle. The parameters of the Boost circuit are set as follows: *L* = 4 mH, *C*_2_* = *470 uF, *f*_2_ = 30 kHz, and the load is a resistor. The duty cycle is used to control the Boost circuit through K60 computing for switch tube to adjust the PV array output voltage. The duty cycles are proportional to the output voltages. The PV powers are changed as the output voltages are adjusted. A large number of experiments show that the duty cycle of the maximum power point is in the range of 15% to 85%.

**Figure 7:**
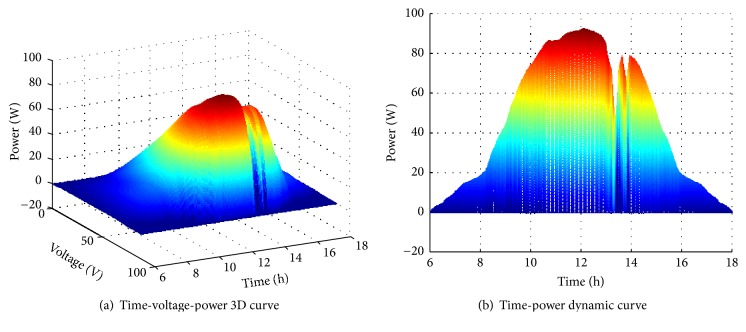
Experimental data.

Experimental data is shown in Figure 7. The experimental time is from 6:00 to 18:00, the PV data of power, voltage, and current were scanned and sampled in 1-minute intervals, and 360 static data curves over time were obtained. The time-voltage-power 3D curve is shown in Figure 7(a). The time-power dynamic curve is shown in Figure 7(b).

**Figure 8:**
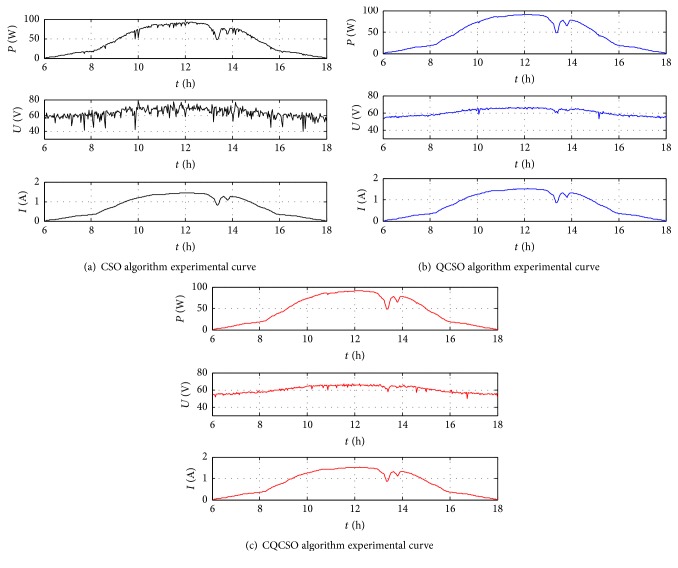
The experiment curve.

The parameters of the CSO, QCSO, and CQCSO algorithm are as follows: the total number of cats is 10, the mixture ratio (MR) is 0.2, the size of seeking memory pool (SMP) in the search group is 3, and the number of tracking loops in the tracking group is 3. Three kinds of algorithms are used to track the 360 data curves, and 360 dynamic maximum power points with time change are obtained as shown in Figure 8. It can be seen that the three algorithms can be very good in tracking the maximum power point in accordance with the environment changes. Between 13:00 and 14:00 on the test day, due to the shelter of the clouds, light radiation decreased and the maximum power values tracked were reduced. Comparing Figures 8(a), 8(b), and 8(c), we find that the QCSO algorithm is the best, the CQCSO algorithm is the second best, and the problem of “premature convergence” in CSO algorithm is serious.

**Figure 9:**
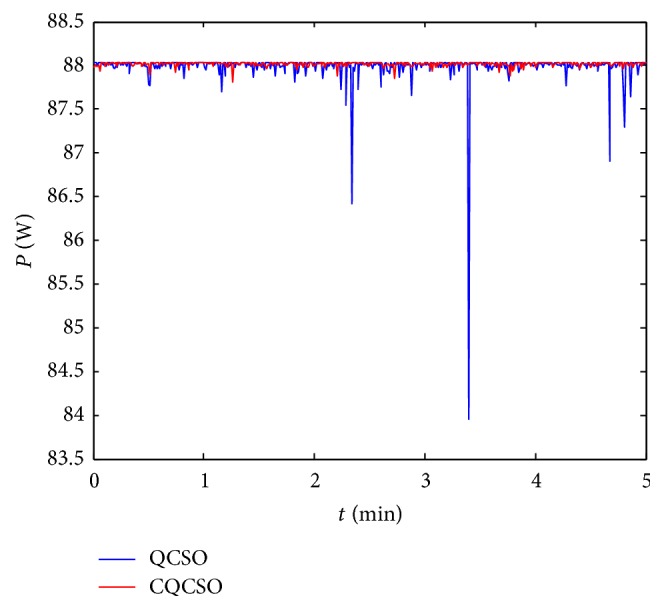
The experiment curve in a complex situation.

In Figure 8, the* P-V* curves are single-peak state under uniform illumination, while the previous simulation and function tests are performed in the multipeak state. Therefore, we simulate complex conditions for the PV system by artificially shading the PV cells. The parameters of the QCSO and CQCSO algorithms are consistent with Figure 8. The experimental test time is 5 minutes. The result is shown in Figure 9.

The experiment results show that the QCSO algorithm has obvious advantages over the CSO algorithm both in the case of a single peak and in the case of multiple peaks. The CQCSO algorithm solves the problem of “premature convergence” in CSO algorithm, but if the PV array is in the uniform illumination condition, the* P-V* curve shows a single-peak state. If the chaos is introduced at this time, although the complexity of the algorithm is increasing, the tracking accuracy is not improved. The QCSO algorithm appears to be better than the CQCSO algorithm, as shown in Figure 8. However, the* P-V* curve is in a multipeak state; it can be seen from Figure 9 which average maximum power value of the CQCSO algorithm is better than the QCSO algorithm in 5 minutes. Therefore, it is concluded that the CQCSO algorithm is more accurate than the QCSO algorithm in the complex case; that is, the QCSO algorithm improved by the tent map has more advantages in solving the multiextremum problem.

## 4. Conclusion

In multilocal extremum optimization, the traditional Cat Swarm Optimization (CSO) algorithm has problems such as “precocity convergence,” slow tracking speed, and poor tracking accuracy. In this paper, we can get the following through the simulation and the experiment.

(1) Firstly, the quantum Cat Swarm Optimization (QCSO) algorithm is proposed. Secondly, Chaos Quantum-behaved Cat Swarm Optimization (CQCSO) algorithm is proposed by introducing tent map. Finally, the CQCSO algorithm is verified by five nonlinear test functions. The simulation results show that the CQCSO algorithm can jump out of the local extreme points and improve the tracking precision and convergence speed.

(2) The CQCSO algorithm is applied to the multipeak maximum power point tracking for photovoltaic array under complex conditions. Both the simulation and experiment results show that the proposed CQCSO algorithm has higher tracking efficiency than the QCSO, CSO, PSO, and CPSO algorithm. In the maximum power point tracking system for photovoltaic power generation, it is certain that it will obtain a larger power value in a shorter period of time, to improve the photovoltaic power generation efficiency.

## Figures and Tables

**Table 1:** Function simulation data.

Algorithm		CSO	QCSO	CQCSO
Schaffer	TIME/s	0.0193	0.0161	0.0156
	BEST	0.0171	0.0147	0.0095
	STD	0.0137	0.0098	0.0017

Shubert	TIME/s	0.2077	0.1776	0.1756
	BEST	−154.4	−166.2	−182.2
	STD	46.350	28.700	4.721

Griewank	TIME/s	0.0317	0.0254	0.0242
	BEST	18.040	5.729	2.987
	STD	8.6060	4.2070	1.5040

Rastrigrin	TIME/s	0.7721	0.6363	0.6343
	BEST	1.3750	0.7618	0.2455
	STD	1.2450	0.5724	0.2701

Rosenbrock	TIME/s	0.0393	0.0338	0.0331
	BEST	26.450	16.710	12.620
	STD	33.250	15.480	6.048

**Table 2:** Parameters of PV modules.

Maximum power value	10 W ±3%	Pressure value of system	1000 VDC
Maximum power voltage	17.5 V	Maximum power current	0.57 A
Open circuit voltage	21.6 V	Short circuit current	0.62 A

**Table 3:** Result of experimental data.

Algorithm	CSO	QCSO	CQCSO	PSO	CPSO
Time/s	0.00291	0.00282	0.00268	0.00431	0.00402
Min/W	79.02	87.38	87.46	73.21	83.1
Max/W	87.95	87.95	88.06	87.94	87.94
Mean/W	87.72	87.89	88.02	86.91	87.66
STD	1.036	0.08009	0.07054	3.03	0.7371
